# Acute inflammatory demyelinating polyneuropathy after treatment with pegylated interferon alfa-2a in a patient with chronic hepatitis C virus infection: a case report

**DOI:** 10.1186/1752-1947-6-278

**Published:** 2012-09-04

**Authors:** Mounia Lahbabi, Meryem Ghissassi, Faouzi Belahcen, Sidi Adil Ibrahimi, Nouredine Aqodad

**Affiliations:** 1 Gastroenterology Unit, Hospital University Hassan II, Fez, Morocco; 2 Neurology Unit, Hospital University Hassan II, Fez, Morocco

**Keywords:** Inflammatory demyelinating polyneuropathy, Neuropathy, PEGylated interferon, Viral hepatitis C infection

## Abstract

**Introduction:**

The combination of polyethylene glycol (PEG)ylated interferon (pegylated interferon) and ribavirin has been shown to be an effective treatment for chronic hepatitis C virus. In general, common side effects related to this combination therapy are mild and are well tolerated. However, peripheral neuropathy including demyelinating polyneuropathy related to PEG-interferon α2a (pegylated interferon alfa-2a) is extremely rare. In the literature, only one case of acute inflammatory demyelinating polyneuropathy related to PEG-interferon α2a has been published previously.

**Case presentation:**

To the best of our knowledge we present only the second case of acute inflammatory demyelinating polyneuropathy related to PEG-interferon α2a, occurring in a 63-year-old Caucasian man. He developed tingling, numbness, and weakness of his upper and lower extremities with acute neurological deficits after five weeks of a combination therapy with PEG-interferon α2a and ribavirin for chronic hepatitis C virus infection. His clinical course, neurological findings, and his electromyogram results were all consistent with acute inflammatory demyelinating polyneuropathy. Our patient recovered completely after interferon was stopped and symptomatic treatment and a further electromyogram showed a disappearance of neuropathy. Four weeks later, PEG-interferon α2a was reintroduced with a gradually increasing dose without any reappearance of neurological symptoms allowing hepatitis C seroconversion.

**Conclusions:**

Recognition of this rare yet possible presentation is important for early and accurate diagnosis and treatment. This case report also suggests that the reintroduction of PEGylated interferon in patients who had presented with acute inflammatory demyelinating polyneuropathy related to interferon α may be safe, but this must be confirmed by further studies.

## Introduction

Interferon α (IFNα) has been widely used for the treatment of chronic viral hepatitis. Overall, the common side effects associated with this drug are well known. The common side effects of IFN include flu-like symptoms, and psychiatric symptoms such as depression, suicidal ideation, irritability, nervousness, and insomnia [1]. Less common side effects include hematopoietic suppression, reversible hair loss, hearing loss, retinopathy, dermatitis, seizures, and the development or exacerbation of autoimmune diseases such as thyroid dysfunction, rheumatoid arthritis, sarcoidosis, systemic lupus erythematosus, vitiligo, type 1 diabetes, and myasthenia gravis [1]. Peripheral neuropathy is a rare and uncommon side effect seen in patients treated with IFNα [2]. In recent years, a variety of peripheral neuropathies have been reported in patients treated with IFN including sensory neuropathy, autonomic neuropathy, Bell’s palsy, and more recently, chronic inflammatory demyelinating polyneuropathy (CIDP) [3]. To date, only one case of acute inflammatory demyelinating polyneuropathy (AIDP) related to polyethylene glycol (PEG)ylated interferon alfa-2a (PEG-IFNα2a) has been published. To the best of our knowledge, this is only the second case of severe AIDP related to PEG-IFNα2a, which developed at week five of IFNα2a therapy initiated for hepatitis C virus infection and where the gradual reintroduction of IFN was safe and without any residual neuromuscular deficits.

## Case presentation

A 63-year-old Caucasian man presented to our hospital for chronic hepatitis C treatment. His initial physical examination results were unremarkable. An initial liver profile included aspartate aminotransferase (AST) 60IU/mL, alanine aminotransferase (ALT) 74IU/mL, total bilirubin 1mg/dL, alkaline phosphatase 81IU/mL, albumin 5.2g/dL, and a prothrombin time (PT) of 76%. Serum viral load was estimated to 3,545,770IU/mL (6.5log) with a genotype 2a2c. The assessment of liver fibrosis by FibroTest® and FibroScan® revealed a stage F2 of fibrosis. An abdominal ultrasound did not find any signs of portal hypertension or cirrhosis. Upper gastrointestinal endoscopy did not find any endoscopic signs of portal hypertension. He was started on a course of treatment comprising PEG-interferon α2a (PEGASYS®) 180μg subcutaneously per week and ribavirin 800mg/day in divided doses orally. A rapid virological response was obtained after four weeks.

In the fifth week of treatment, he reported painful paresthesia, tingling, numbness, and weakness of his lower extremities. His neurological symptoms progressed rapidly over the course of one week to the point of losing the ability to ambulate. In addition, our patient also complained of tingling and numbness in the upper extremities involving the fingers and wrists bilaterally. PEGASYS® was discontinued due to an acute onset of weakness of the upper and lower extremities. A neurological examination revealed normal cranial nerves with normal bulk of muscles and tone; but weak muscle strength in the upper extremities as well as in the hips, knees, ankles and toes bilaterally. His tendon reflexes were diminished significantly. A sensory examination revealed decreased light touch, pinprick, and temperature sensation from the feet to mid-calves as well as hands, except for vibratory sense. A clinical diagnosis of acquired acute inflammatory demyelinating sensorimotor polyneuropathy was made. Biochemical screening tests, complete blood count, erythrocyte sedimentation rate, anti-nuclear antibodies, serum cryoglobulin level, human immunodeficiency virus (HIV) test and cytomegalovirus (CMV) deoxyribonucleic acid (DNA) were all normal. The results of an electromyogram (EMG) revealed evidence of predominant motor polyneuropathy with prominent demyelinating features, including motor conduction block, low conduction velocity, and prolonged minimum F wave latency in the bilateral ulnar, median, peroneal, and tibial nerves (Table 1). Our patient recovered completely after one month of stopping interferon (without stopping ribavirin) and symptomatic treatment. His muscle weakness and paresthesia were markedly improved, with normal strength in all muscle groups; eventually he could walk without difficulty. A further EMG showed a disappearance of neuropathy. Four weeks later, IFN was reintroduced with a gradually increasing dose (90μg for two months, then 120μg for one month followed by 180μg for the two remaining months) without reappearance of neurological symptoms. He has not experienced relapse and his neurological status remained stable. His viral load of hepatitis C was negative at week 24 and six months after stopping treatment. Currently our patient is well and his neurological symptoms have resolved completely with no residual neuromuscular deficits.

**Table 1:** Electromyogram/nerve conduction studies

	**Distal latency (m/s)**	**Conduction velocity (m/s)**	**Amplitude (uV (sensory) mV (motor))**
**Sensory**			
Right median	3.2	48	4.1
Right ulnar	2.3	46	1
Right sural	2.8	40	3
**Motor**			
Right median	4.1	29.4	2.4
Right ulnar	6.2	38.1	1
Right tibial	7.1	30.9	1.3
Right peroneal	8.2	22.8	0.5
**F wave**	**Minimum F-wave latency (ms)**		
Right median	53.6		
Right ulnar	43.2		
Right tibial	50		
Right peroneal	56.9		

## Discussion

The more probable etiologies of demyelinating polyneuropathy in our patient included infectious processes, toxins and/or drugs, and immune mediated processes [4]. Viral infections such as hepatitis C virus (HCV), HIV and CMV have been described to cause demyelinating neuropathy [5]. Since polyneuropathy developed after the negative conversion of HCV-ribonucleic acid (RNA) transcript, and both HIV test and CMV DNA results were negative in our patient, these were not the cause of his neuropathy. Toxins and/or drugs are also common etiologies for demyelinating neuropathies, which can be acute or chronic in nature [4]. Common toxins/drugs are barbital, sulfonamides, phenytoin, nitrofurantoin, heavy metals, carbon monoxide, industrial poisons, and certain drugs used for acquired immunodeficiency syndrome (AIDS) (zalcitabine, didanosine) [4]. Our patient had no exposure to any of these toxins or drugs other than PEGASYS® and ribavirin. Indeed, ribavirin has not been associated with any type of reported neuropathy, but PEGASYS® including IFNα has been implicated to cause immune-mediated CIDP during chronic hepatitis C treatment due to cytokine-induced apoptosis in the myelin-producing oligodendrocytes, resulting in inhibition of central nervous system remyelination [6]. The timecourse for our patient’s symptoms was not long enough to meet the criteria for a CIDP. The criteria for CIDP usually include the clinical deterioration of neurological symptoms for a period greater than eight weeks, as opposed to AIDP, which usually has deterioration over a period of approximately four weeks or less as in our patient’s case [7]. The pathogenesis of AIDP related to PEGASYS® use is thought to be an acute immune-mediated progress. The precise and exact immune regulatory function of IFNα including PEG-IFN is not well understood, and it is likely similar to the mechanism associated with IFN-induced CIDP described above [3,8]. In addition, IFNα has been reported to enhance *in vivo* and *in vitro* autoantibody production and may upregulate transcription of genes associated with class I major histocompatibility complex (MHC) antigens [9]. It is likely that the levels of pro-inflammatory cytokines may trigger autoimmune phenomena in immunologically predisposed individuals when IFN is administered. Therefore, the immune system mistakenly attacks the host’s nerve tissue after recognizing a molecular epitope similar to a foreign antigen and this may result in acute inflammatory neuropathy [10]. In addition, there have been multiple reported cases of demyelinating neuropathy associated with cyroglobulinemia related to HCV [11]. However, our patient had an undetectable cryoglobulin level on multiple occasions, which rules out a possibility of cryoglobulin-induced demyelinating neuropathy. Peripheral neuropathy related to PEGASYS® improves after stopping treatment; some cases may require the use of corticosteroids, plasmapheresis or immunosuppressive drugs [12]. In our patient’s case his symptomatology improved significantly four weeks after stopping interferon and the use of a symptomatic treatment. Four weeks later, IFN was reintroduced with a gradually increasing dose without reappearance of his neurological symptoms.

## Conclusions

Acute inflammatory demyelinating neuropathy is a rare complication of IFNα therapy. It is very important that gastroenterologists and hepatologists recognize this rare disorder for early diagnosis, management, and the prevention of long-term neurological deficits. This case report also suggests that the reintroduction of PEGylated interferon may be safe, but this must be confirmed by further studies.

## Consent

Written informed consent was obtained from the patient for publication of this case report and any accompanying images. A copy of the written consent is available for review by the Editor-in-Chief of this journal.

## Competing interests

The authors declare that they have no competing interests.

## Authors’ contributions

LM, AN and ISA analyzed and interpreted the data from our patient and wrote the manuscript. GM and BF performed the nerve conduction studies, and were major contributors in writing the manuscript. All authors have read and approved the final manuscript.
